# Plasma β-Amyloid Levels Associated With Structural Integrity Based on Diffusion Tensor Imaging in Subjective Cognitive Decline: The SILCODE Study

**DOI:** 10.3389/fnagi.2020.592024

**Published:** 2021-01-12

**Authors:** Xiaoni Wang, Mingyan Zhao, Li Lin, Ying Han

**Affiliations:** ^1^Department of Neurology, Xuanwu Hospital of Capital Medical University, Beijing, China; ^2^National Clinical Research Center for Geriatric Disorders, Beijing, China; ^3^Center of Alzheimer's Disease, Beijing Institute for Brain Disorders, Beijing, China

**Keywords:** plasma β-amyloid, diffusion tensor imaging, subjective cognitive decline, white matter, blood-based biomarker

## Abstract

**Background:** Accumulating evidence has demonstrated that plasma β-amyloid (Aβ) levels are useful biomarkers to reflect brain amyloidosis and gray matter structure, but little is known about their correlation with subclinical white matter (WM) integrity in individuals at risk of Alzheimer's disease (AD). Here, we investigated the microstructural changes in WM between subjects with low and high plasma Aβ levels among individuals with subjective cognitive decline (SCD).

**Methods:** This study included 142 cognitively normal individuals with SCD who underwent a battery of neuropsychological tests, plasma Aβ measurements, and diffusion tensor imaging (DTI) based on the Sino Longitudinal Study on Cognitive Decline (SILCODE). Using tract-based spatial statistics (TBSS), we compared fractional anisotropy (FA), and mean diffusivity (MD) in WM between subjects with low (*N* = 71) and high (*N* = 71) plasma Aβ levels (cut-off: 761.45 pg/ml for Aβ40 and 10.74 pg/ml for Aβ42).

**Results:** We observed significantly decreased FA and increased MD in the high Aβ40 group compared to the low Aβ40 group in various regions, including the body, the genu, and the splenium of the corpus callosum; the superior longitudinal fasciculus; the corona radiata; the thalamic radiation; the external and internal capsules; the inferior fronto-occipital fasciculus; and the sagittal stratum [*p* < 0.05, familywise error (FWE) corrected]. Average FA values were associated with poor performance on executive and memory assessments. No significant differences were found in either MD or FA between the low and high Aβ42 groups.

**Conclusion:** Our results suggest that a correlation exists between WM integrity and plasma Aβ40 levels in individuals with SCD.

## Introduction

Extracellular β-amyloid (Aβ) accumulation and intracellular tau deposition are the core features of Alzheimer's disease (AD; Jack et al., 2018). Amyloid pathology is defined as the initiating step of AD, which leads to subsequent tau deposition and neurodegeneration (Long and Holtzman, 2019); however, the well-established and validated biomarkers for brain amyloidosis detection, including cerebrospinal fluid (CSF) analysis and amyloid PET, are expensive, invasive, and difficult to implement on a large scale in clinical practice (Sperling et al., 2011, 2014; Dubois et al., 2014; Honig et al., 2018). Therefore, minimally invasive and affordable techniques to support early screening are urgently needed.

Blood-based biomarkers represent a logical alternative. Circulating Aβ peptides are the most studied AD biomarkers in plasma. Growing evidence has demonstrated that plasma Aβ concentrations are highly correlated with brain amyloidosis (Nakamura et al., 2018; Risacher et al., 2019; Schindler et al., 2019; Vergallo et al., 2019; Doecke et al., 2020), and the plasma Aβ42/Aβ40 ratio has an accuracy of over 90% in identifying brain amyloid positivity (Schindler et al., 2019; Doecke et al., 2020). Several studies have also reported an association between plasma Aβ levels and gray matter changes, including gray matter volume and cerebral cortex thickness, in both cognitively normal subjects and subjects with mild cognitive impairment (MCI) and AD-related dementia (Kaffashian et al., 2015; Llado-Saz et al., 2015; Cantero et al., 2016; Hanon et al., 2018; Hilal et al., 2018; Youn et al., 2019), suggesting that plasma Aβ levels may reflect downstream neurodegeneration.

White matter (WM) neurodegeneration of associative fiber tracts in AD may result from gray matter atrophy and Wallerian degeneration (Hardy and Higgins, 1992). Accumulating evidence has demonstrated disrupted WM integrity in patients with AD, MCI, and preclinical AD, which is related to cognitive decline (Mayo et al., 2017; Brueggen et al., 2019; Power et al., 2019). Though studies have shown an association of plasma Aβ levels with WM macrostructures such as lesions, hyperintensities, and atrophy (Janelidze et al., 2016; Hilal et al., 2017; Lippa et al., 2019; Youn et al., 2019), the relationship between plasma Aβ levels and WM microstructure has not been clarified.

Subjective cognitive decline (SCD) refers to those who experience subjective cognitive deficits without measurable cognitive impairment (Jessen et al., 2014, 2020). It is suggested as one of the earliest manifestations of the AD continuum, and accumulating evidence has demonstrated that individuals with SCD may exhibit an increased risk of progression to cognitive impairment and of developing AD (Mitchell et al., 2014; Slot et al., 2019) and may present increased AD pathology (Amariglio et al., 2015). Regardless of the absence of objective cognitive impairment (OCI), SCD might become important for clinical practice as an early trigger for seeking medical help because of an increase in the number of individuals with SCD in the healthcare system (Jessen et al., 2020). Thus, taking individuals with SCD as an interesting target population to study may enhance our understanding of early AD diagnosis and preventive treatment. Recently, several studies have identified the correlation between plasma Aβ and gray matter volume by performing structural magnetic resonance imaging (sMRI) in individuals with SCD (Cantero et al., 2016; Youn et al., 2019). Our previous study showed widespread WM microstructure impairment in SCD (Li et al., 2016); however, studies on its correlation with plasma Aβ in this stage remain lacking.

Diffusion tensor imaging (DTI) can be employed for *in vivo* detection of WM microstructural properties. Fractional anisotropy (FA) and mean diffusivity (MD) are the most commonly used types of indices in AD research, and they reflect microstructural neuronal dysfunctions that precede macroscopic atrophy (Soares et al., 2013; Qin et al., 2020). In this study, we aimed to assess whether plasma Aβ levels are related to subclinical microstructural WM integrity as measured by DTI, and first, we hypothesized that higher plasma Aβ40 and lower Aβ42 levels are associated with WM integrity. Second, we hypothesized that plasma Aβ-related WM impairment is associated with cognitive decline.

## Materials and Methods

### Participants

The baseline dataset of the Sino Longitudinal Study on Cognitive Decline (SILCODE; Li et al., 2019) from March 20, 2017 to September 17, 2018, was included in the study. Excluding all cases that failed to meet the inclusion criteria, a total of 142 cognitively normal elderly Han Chinese subjects with SCD (mean age: 66.07 ± 3.88 years) were included. In addition, 26 patients with MCI and AD-related dementia categorized as patients with OCI in the present study were included for complementary analyses. All participants underwent clinical assessment, a battery of neuropsychological tests, blood sample collections, and MRI scans.

All participants were between 60 and 80 years old. SCD is defined as follows (Jessen et al., 2014): (1) the onset of self-experienced persistent decline (>6 months) within the last 5 years; (2) the onset of subjective decline in memory rather than other domains (language, attention, planning, and any other cognitive decline); (3) participants within the normal range upon cognitive testing (adjusted for age, sex, and education) and failure to meet the criteria for MCI or dementia. MCI was diagnosed if they met any one of the following three criteria (Bondi et al., 2008; Jak et al., 2009): (1) impaired scores (defined as >1 SD below the age-corrected normative means) on both measures in at least one cognitive domain (memory, language, or speed/executive function); (2) impaired scores in each of the three cognitive domains (memory, language, or speed/executive function); and (3) the Functional Activities Questionnaire (FAQ) ≥9. AD-related dementia was diagnosed based on the Diagnostic and Statistical Manual of Mental Disorders, Fourth Edition (DSM-IV) and the National Institute on Aging and the Alzheimer's Association (NIA-AA) workgroup guidelines for dementia due to AD. To eliminate the impact of cerebral vascular disease, we excluded subjects with a history of stroke, large-vessel disease (cortical and/or subcortical infarcts and watershed infarcts), moderate WM changes, and multiple lacunar infarcts (>1) on brain imaging. The SILCODE exclusion criteria ensured that no subjects with current major psychiatric diagnosis; neurological disease; systematic disease that causes cognitive decline, head trauma, or unstable medical conditions were included. All subjects gave their written informed consent prior to participation. The study protocol was approved by the Medical Research Ethics Committee and Institutional Review Board of Xuanwu Hospital. The SILCODE is listed in the ClinicalTrail.gov registry (NCT02225964).

### Neuropsychological Assessments

We performed a battery of neuropsychological tests covering memory, language, and executive function. Auditory Verbal Learning Test - Huashan version (AVLT)-long delayed recall and -recognition (Xu et al., 2020) was used to evaluate memory; Semantic Verbal Fluency Test (VFT; Guo et al., 2007) and Boston Naming Test (BNT; Guo et al., 2006) were administered to evaluate language; and time consumed in Shape Trail Test A (STT-A) and B (STT-B; Zhao et al., 2013) were used to evaluate executive function. The thresholds for memory, language, and executive function tests are summarized in Supplementary Table 1. The SCD questionnaire including nine reliable SCD items (SCD-Q9) was used for the quantitative assessment of the severity of SCD (Gifford et al., 2015). Mini-Mental State Examination (MMSE) and Montreal Cognitive Assessment Basic Version (MoCA-B) were used to evaluate general cognitive ability. Besides, all subjects were assessed with Hamilton Anxiety Scale (HAMA), Hamilton Depression Scale (HAMD), Geriatric Depression Scale (GDS), and the FAQ.

### Plasma Aβ Measurements

Blood samples were collected in the morning after an overnight fast. After centrifugation, the samples were aliquoted, stored at −80°C, and thawed immediately on ice before assaying. Meso Scale Discovery (MSD) kits (Rockville, Maryland, USA) were used to quantify the concentrations of plasma Aβ. All the samples were measured in duplicate using the same aliquot following the manufacturer's instructions. The detection limits were 20–6,000 pg/ml for Aβ40 and 2.5–1,271 pg/ml for Aβ42. The mean inter-assay and intra-assay coefficients of variation were <10 and 6%, respectively, for both Aβ40 and Aβ42. The 142 subjects with SCD were then divided into low and high Aβ groups (*N* = 71 case/group) with the cut-off defined by the mean value (Aβ40: 761.45 pg/ml; Aβ42: 10.74 pg/ml).

### Image Acquisition and Analysis

All MRI data were acquired on an integrated simultaneous 3.0 T TOF PET/MR (Signa PET/MR, GE Healthcare, Milwaukee, WI, USA) at Xuanwu Hospital of Capital Medical University. DTI scans were collected axially with a single-shot spin-echo diffusion-weighted echo planar imaging (EPI) sequence. The parameters were as follows: 30 gradient directions and 5 b0 images (b = 1,000 s/mm^2^), field of view (FOV) = 256 × 256 × 256, matrix = 112 × 112, repetition time = 16,500 ms, echo time = 95.6 ms, slice number = 70, slice thickness = 2 mm, and voxel size = 2 × 2 × 2 mm^3^. Three-dimensional T1 weighted images were acquired with a Spoiled Gradient Recalled Echo (SPGR) sequence. Additionally, T2 weighted and resting-state functional MR images were collected. The parameter details have been described in previous studies (Li et al., 2019; Sun et al., 2019; Dong et al., 2020).

The DTI data of each subject were processed with a pipeline tool for analyzing brain diffusion images (PANDA; Cui et al., 2013), which integrates the FMRIB Software Library (FSL; Smith et al., 2004), the Pipeline System for Octave and Matlab (PSOM; Bellec et al., 2012), the Diffusion Toolkit, and the MRIcron. The main steps of data preprocessing were as follows: (1) converting DICOM files into NIFIT format; (2) estimating the brain mask: The *bet* command of FSL was used to remove the skull from b0 image; (3) cropping the raw image: The *fslroi* command of FSL was used to remove non-brain tissue; (4) correcting for the eddy-current effect: Head motion and eddy current distortions were corrected by registration of the diffusion-weighted images to the b0 images using the *eddy_correct* command of FSL; and (5) calculating diffusion tensor parameters: The *dtifit* command of FSL was applied to calculate FA and MD maps. Tract-based spatial statistics (TBSS) were performed for the voxel-wise analysis of FA and MD (Smith et al., 2006). All individual images were registered to the 1 × 1 × 1 mm Montreal Neurological Institute (MNI) standard space with the FMRIB58_FA template as the target image (http://www.fmrib.ox.ac.uk/fsl/data/FMRIB58_FA). Then, a mean FA average was obtained by averaging the FA images from each subject in the standard space and thinning to create a custom mean FA skeleton. The mean FA skeleton was thresholded at 0.2 to include only voxels indicative of WM. Then, the individual FA maps were projected onto the FA skeleton to obtain the FA skeletons of each participant and the deformation matrixes. This projection information was also applied to MD. The skeletonized FA and MD maps were used in further statistical analysis.

### Statistical Analysis

Differences between the low and high Aβ groups in demographic data and vascular comorbidity distribution were compared using the two-sample *t*-test for continuous variables and the chi-square test for categorical variables. To compare cognitive functions, the general linear model (GLM) controlling for age, sex, and years of education was conducted with neuropsychological tests as independent variables and Aβ groups as dependent variables.

Voxel-wise cross-subject comparisons were performed using the randomize tool in FSL, which is used for non-parametric permutation-based testing. FA and MD were compared through a GLM with Aβ groups as dependent variables. The design matrix included age, sex, and years of education as nuisance covariates. Significant differences were estimated with 5,000 random permutations using threshold-free cluster enhancements (TFCE) and FWE correction for multiple comparisons. The significance threshold was *p* < 0.05 and voxels > 100 (TFCE and FWE corrected). Then, the significant results were thickened with the tbss_fill tool in FSL for better visualization. Finally, the John Hopkins University (JHU) White-Matter Tractography Atlas and JHU-ICBM-DTI-81 White-Matter Labels Atlas were used to identify regions of statistical significance (Mori et al., 2008). Complementary analyses were conducted using the plasma Aβ levels as continuous variables and by assessing subclinical WM integrity correlations. The correlations were run separately in the SCD and OCI groups.

To determine the relationships between the WM parameters and cognitive function, partial correlation analysis controlling for age, sex, and year of education was performed between the impaired cognitive scores and average FA (MD) values of regions showing significant group differences. The significance threshold was *p* < 0.05.

## Results

### Behavioral Results

Table 1 summarizes the demographic and neuropsychological results according to plasma Aβ levels in SCD. Age, sex, years of education, ApoE genotype distribution, and vascular comorbidities were statistically homogeneous. The high Aβ40 group exhibited poorer performance on memory, executive, and language tests (AVLT-DR: F = 4.652, *p* = 0.033; AVLT-R: F = 7.219, *p* = 0.008; STT-A: F = 4.271, *p* = 0.0341; VFT: F = 5.260, *p* = 0.023). The low and high Aβ42 groups showed no significant difference in cognitive tests in the three domains. No significant differences in SCD-Q9 scores between the low and high Aβ groups were detected (neither Aβ40 nor Aβ42). The demographic and neuropsychological results in the total SCD sample and the OCI sample are summarized in Supplementary Table 2.

**Table 1:** Demographic and neuropsychological results.

	**Aβ40**		***p***	**Aβ42**		***p***
	**Low**	**High**		**Low**	**High**	
Age	65.68 ± 3.64	66.45 ± 4.10	0.241	66.67 ± 3.92	66.47 ± 3.83	0.215
Sex (M/F)	23/48	22/49	0.857	22/49	23/48	0.857
Education	12.51 ± 2.94	11.96 ± 3.01	0.279	12.25 ± 2.76	12.22 ± 3.18	0.944
ApoE ε4 carrier, n%	21 (29.6)	14 (19.7)	0.173	14 (19.7)	21 (29.6)	0.173
Hypertension, n%	26(36.7)	30 (42.3)	0.492	32 (45.1)	24 (33.8)	0.170
Diabetes, n%	11 (15.5)	9 (12.7)	0.629	13 (18.3)	7 (9.6)	0.148
Hyperlipidemia, n%	27 (38.0)	26 (36.6)	0.862	27 (38.0)	26 (36.6)	0.862
Smoking, n%	15 (21.1)	14 (19.7)	0.835	16 (22.5)	13 (18.3)	0.532
SCD-9	4.80 ± 1.70	4.68 ± 1.91	0.607	4.52 ± 1.63	5.03 ± 1.94	0.057
AVLT-DR	7.56 ± 1.93	6.73 ± 2.20	0.033	6.89 ± 1.87	7.41 ± 2.30	0.088
AVLT-R	22.72 ± 1.40	21.096 ± 1.74	0.008	22.27 ± 1.68	22.27 ± 1.68	0.697
STT-A	57.73 ± 15.29	64.20 ± 16.43	0.041	61.40 ± 16.68	60.52 ± 15.71	0.636
STT-B	134.18 ± 34.16	139.89 ± 31.52	0.564	141.24 ± 33.68	132.83 ± 31.73	0.060
VFT	19.79 ± 4.45	17.89 ± 4.29	0.023	19.14 ± 4.55	18.54 ± 4.38	0.470
BNT	25.30 ± 2.74	24.63 ± 2.96	0.223	24.83 ± 3.02	25.10 ± 2.71	0.628
MMSE	28.93 ± 1.18	28.59 ± 1.72	0.285	28.68 ± 1.32	28.85 ± 1.63	0.395
MoCA-B	25.97 ± 2.47	25.37 ± 2.15	0.218	25.41 ± 2.20	24.93 ± 2.44	0.117
GDS	2.41 ± 2.00	2.94 ± 2.61	0.174	2.54 ± 2.21	2.83 ± 2.46	0.358
HAMA	4.37 ± 3.17	4.56 ± 3.93	0.856	4.30 ± 3.56	4.63 ± 3.58	0.580
HAMD	3.97 ± 3.95	4.37 ± 8.39	0.438	4.21 ± 4.34	4.13 ± 3.46	0.957
FAQ	0.18 ± 0.49	0.28 ± 0.83	0.46	0.20 ± 0.50	0.27 ± 0.83	0.587

*Values of p for neuropsychological tests were obtained with the general linear model adjusted for age, sex, and years of education. ApoE, apolipoprotein E; AVLT-DR, Auditory Verbal Learning Test-long delayed recall; AVLT-R, Auditory Verbal Learning Test-recognition; STT-A Shape Trail Test A; STT-B, Shape Trail Test B; VFT, Verbal Fluency Test; BNT, Boston Naming Test; MMSE, Mini-Mental State Examination; MoCA-B, Montreal Cognitive Assessment Basic Version; GDS, Geriatric Depression Scale; HAMA, Hamilton Anxiety Scale; HAMD, Hamilton Depression Scale; FAQ, Functional Activities Questionnaire*.

### Comparisons of Whole Brain WM Between the Low and High Aβ Groups

**Figure 1:**
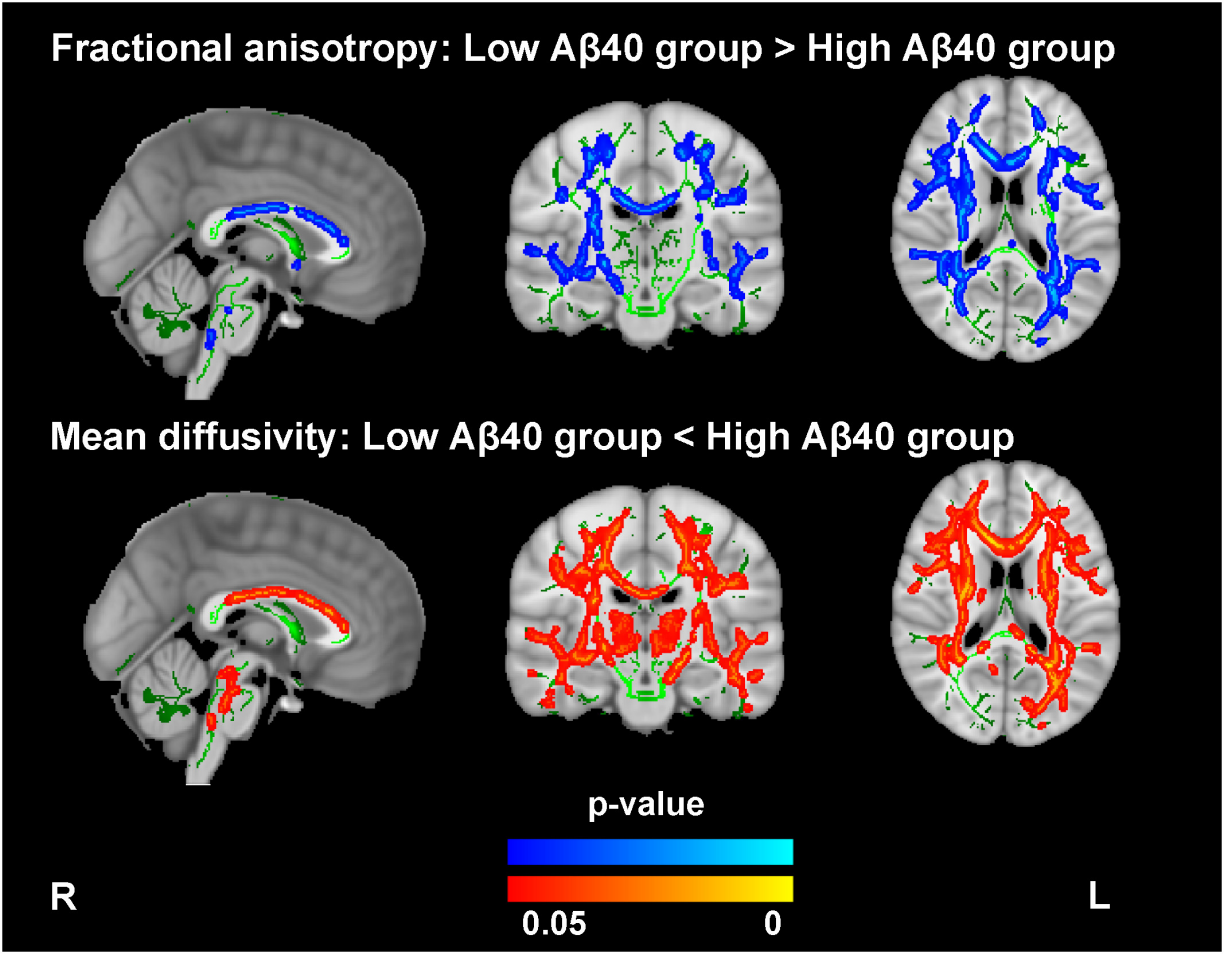
Comparison of the fractional anisotropy (FA) and mean diffusivity (MD) findings between the high and low Aβ40 groups. The averaged skeleton (green color) was overlaid with significantly lower FA (blue-light color) and higher MD (red-yellow color) in the high Aβ40 group compared with the low Aβ40 group (TFCE and FWE corrected *p* < 0.05, voxels > 100). The analysis controlled for age, sex, and years of education.

Compared with the low Aβ40 group, the high Aβ40 group exhibited decreased FA and increased MD in widespread WM tracts (TFCE and FWE corrected, *p* < 0.05), mainly located in the body, the genu, and the splenium of corpus callosum; the superior longitudinal fasciculus; the anterior, superior, and posterior corona radiata; the thalamic radiation; the external and internal capsules; the inferior fronto-occipital fasciculus; the sagittal stratum; the cerebral peduncle; and the fornix (see Figure 1 and Supplementary Table 3). After FWE correction, no significant differences were noted for FA and MD between the low and high Aβ42 groups.

### Relationship Between WM and Neuropsychological Tests

**Figure 2:**
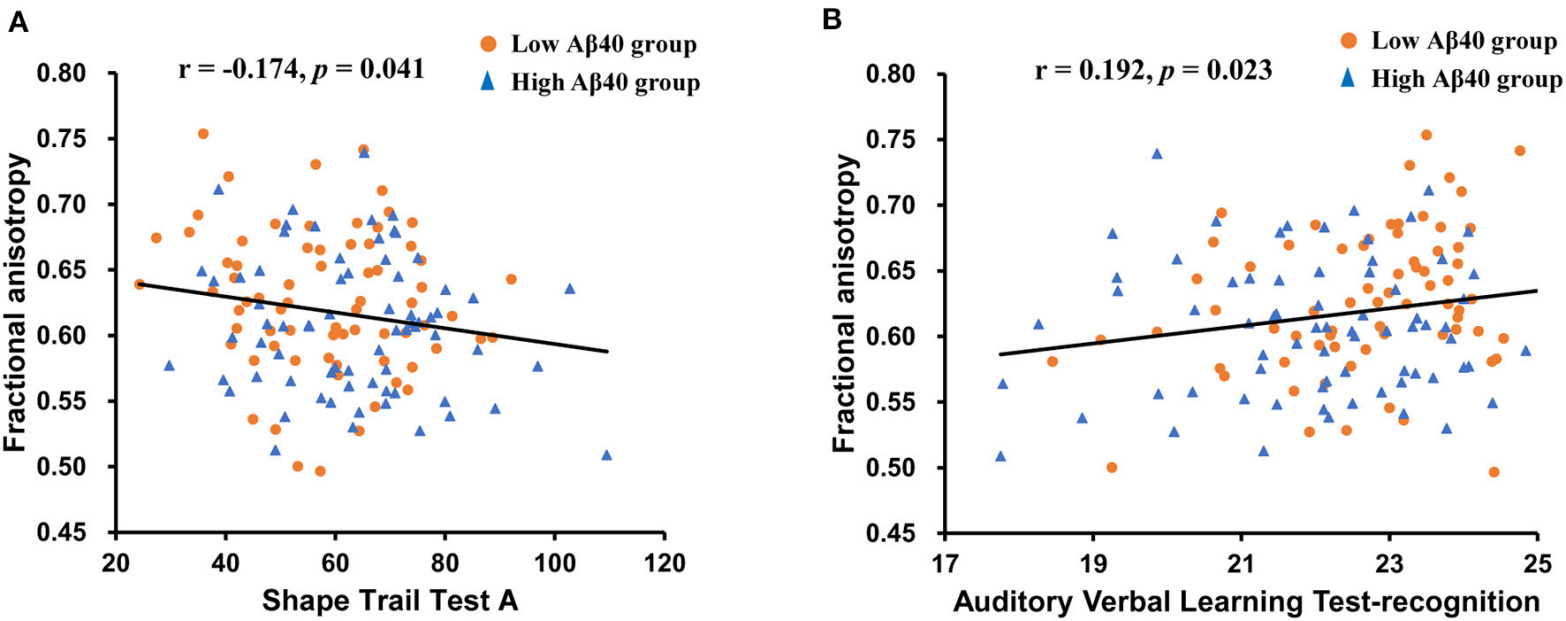
Scatter plots illustrating the relationships between average white matter (WM) parameters and neuropsychological tests controlling for age, sex, and years of education. **(A)** A significant negative correlation was found between the FA values and the Shape Trail Test-A (STT-A) scores. **(B)** A significant positive correlation was found between FA values and Auditory Verbal Learning Test (AVLT)-recognition scores.

In the extracted cluster, the relationship between average MD and FA values and impaired cognitive tests (AVLT-DR, AVLT-R, STT-A, and VFT) observed in the high Aβ40 group was investigated. Age, sex, and years of education were included as covariates. Average FA values were negatively correlated with STT-A (*r* = −0.174, *p* = 0.041) and positively correlated with AVLT-R (*r* = 0.192, *p* = 0.023; see Figure 2). No significant correlation between MD values and cognitive scores was noted (see Supplementary Table 4).

### Complementary Analysis

**Figure 3:**
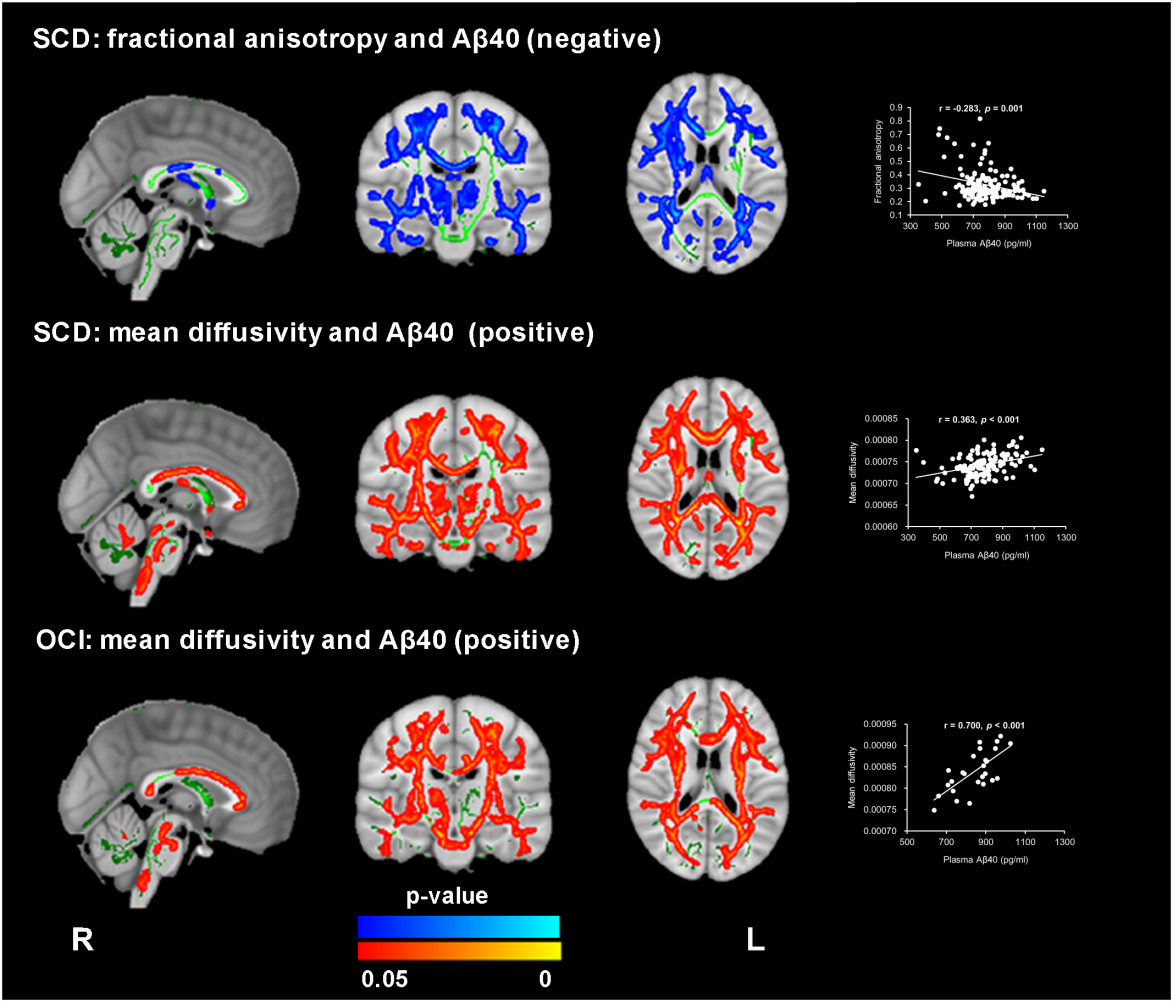
Significant association between WM microstructure parameters and plasma Aβ40 in subjective cognitive decline (SCD) and patients with objective cognitive impairment (OCI). FA revealed a negative correlation with plasma Aβ40 in SCD (blue-light color), and MD exhibited a positive correlation with plasma Aβ40 in both the SCD and OCI groups (red-yellow color) (TFCE and FWE corrected *p* < 0.05, voxels > 100). The regions with statistical significance were projected on the averaged skeleton (green color). The scatter plots show the average values (y-axis) from the significant regions for each subject against plasma Aβ40 (x-axis) for each participant. The analysis controlled for age, sex, and years of education.

In the SCD group, the voxel-wise analysis using the plasma Aβ levels as continuous variables revealed a significant association of higher plasma Aβ40 levels with decreased FA and increased MD values (TFCE and FWE corrected, *p* < 0.05), which were located in similar regions as noted in the group comparison (see Figure 3 and Supplementary Table 5). In the OCI group, a positive correlation between MD and plasma Aβ40 levels was identified in the bilateral forceps minor, the superior longitudinal fasciculus, the inferior longitudinal fasciculus, the anterior thalamic radiation, the inferior fronto-occipital fasciculus, the cingulum, and the corticospinal tract (see Figure 3 and Supplementary Table 5). Both correlations between Aβ42 and FA and MD were not significant within the SCD and OCI groups.

## Discussion

In the present study, we investigated the association between plasma Aβ levels and WM microstructure. Both decreased FA and increased MD values were found in the high Aβ40 group and were mainly located in the corpus callosum, the superior longitudinal fasciculus, the corona radiata, the thalamic radiation, the external and internal capsules, the inferior fronto-occipital fasciculus, the sagittal stratum, the cerebral peduncle, and the fornix. Moreover, the decreased FA was associated with poor performance on STT-A and AVLT-R; however, no significant differences were found in the DTI parameters between the low and high Aβ42 groups.

This study identified an association between higher plasma Aβ40 levels and WM microstructure abnormalities in individuals with SCD and OCI. Though some studies have investigated the association between plasma Aβ40 levels and WM hyperintensities, lesions and WM volume (Janelidze et al., 2016; Hilal et al., 2017; Youn et al., 2019), we found that the correlation between the plasma Aβ40 levels and WM integrity remained significant after excluding those with cerebral vascular disease. Our results were consistent with previous studies that showed the association between plasma Aβ40 levels and neurodegeneration biomarkers, such as hippocampal atrophy and thinner cerebral cortex thickness, in both cognitively normal elderly subjects and patients with AD (Kaffashian et al., 2015; Llado-Saz et al., 2015; Hanon et al., 2018). The association between plasma Aβ40 and WM integrity may result from the role of Aβ40 in cerebrovascular abnormalities in AD. Aβ40 was found to reproduce the cerebrovascular alterations in transgenic mice overexpressing the amyloid precursor protein (APP; Niwa et al., 2000), and circulating Aβ40 could enhance the cerebrovascular dysfunction induced by brain Aβ40, which may contribute to WM impairment (Park et al., 2013). Though the age was matched between the low and high Aβ40 groups and included as a covariate for statistical analysis, we cannot exclude the possibility that the association of age with both plasma Aβ40 levels and DTI parameters contributed to the correlation found in our study (Kleinschmidt et al., 2016; Lovheim et al., 2017; Jiang et al., 2019; Zavaliangos-Petropulu et al., 2019).

The correlations with Aβ40 seemed to be more widespread and pronounced in MD compared with FA in both the SCD and OCI groups. The biological bases of MD and FA may differ and are not fully understood. FA corresponds to the degree of directionality and anisotropic diffusion, which is assumed to reflect WM impairment caused by microstructural damage such as axonal degradation (Soares et al., 2013; Brueggen et al., 2019). In contrast, MD is calculated based on the mean of three eigenvalues and corresponds to the diffusion rate, which is assumed to reflect WM impairment caused by membrane integrity damage. FA analysis can be influenced by crossing fibers more than MD, which may limit its power to detect WM degeneration (Soares et al., 2013; Brueggen et al., 2019).

Contrary to our hypothesis, we did not identify significant differences in FA and MD between the low and high Aβ42 groups, where the identification is consistent with a previous study in patients with a history of traumatic brain injury (Lippa et al., 2019). Peripheral Aβ42 is highly correlated with brain amyloid pathology (Nakamura et al., 2018; Schindler et al., 2019; Doecke et al., 2020); whereas, several studies have indicated that the loss of WM integrity reflects early tau accumulation other than amyloid pathology (Strain et al., 2018). Kantarci et al. (2017) reported higher MD and lower FA in higher Braak neurofibrillary tangle staging than in those with high Aβ neuritic plaques, which may result in the lack of association between WM parameters and plasma Aβ42 in our study. Our results indicated that plasma Aβ42 levels may not reflect subclinical WM impairment.

We observed significant differences between the low and high Aβ40 groups in memory, executive, and language domains. Subjects with increased Aβ40 performed poorly on cognitive tests, though the performance of the participants in the present study on cognitive tests was within the normal range. Consistent with our findings, large population-based studies have reported the association of increased Aβ40 with the risk of dementia as well as with declining cognitive measurements (Hilal et al., 2018; Verberk et al., 2018); however, some studies also found an association between plasma Aβ42 and impaired cognition (Llado-Saz et al., 2015). Differences in patient age, clinical status, and analysis techniques may affect plasma Aβ quantification and result in inconsistencies between studies (Toledo et al., 2013; Palmqvist et al., 2018; Wang et al., 2018). We further found an association between the STT-A and AVLT-R scores, and the average FA values within the regions exhibit significant group differences, indicating that plasma Aβ40-related WM structural changes may reflect cognitive function in individuals with SCD.

Our study has several limitations. First, this study employed a cross-sectional design. We found that higher Aβ40 levels were associated with disrupted diffusion in WM; however, whether the plasma Aβ40 level correlated with the cause of WM abnormalities in subjects with SCD was not clarified. The lack of repeated measurements of blood Aβ concentrations limits the evaluation of the trajectory of plasma levels in relation to WM impairment. Thus, longitudinal studies are needed to identify the dynamic correlation between plasma Aβ levels and WM integrity. Second, we did not analyze brain amyloid or tau pathology; therefore, further studies are needed to determine whether central amyloid or tau induces the association between plasma Aβ levels and WM integrity. Finally, additional studies should be conducted in subjects with different cognitive statuses to determine whether the correlations are dependent on disease progression.

## Conclusion

In summary, the current study demonstrated different WM microstructures between subjects with low and high Aβ40 levels among individuals with SCD. The findings suggest that plasma Aβ40 levels could reflect central neurodegeneration and may represent a useful biomarker to predict different trajectories of aging in individuals with SCD.

## Data Availability Statement

The raw data supporting the conclusions of this article will be made available by the authors, without undue reservation.

## Ethics Statement

The studies involving human participants were reviewed and approved by Medical Research Ethics Committee and Institutional Review Board of Xuanwu Hospital. The patients/participants provided their written informed consent to participate in this study.

## Author Contributions

XW and YH did manuscript preparation and drafting. XW, MZ, LL, and YH did the clinical assessments and data acquisition. MZ and YH did the clinical diagnosis. XW, MZ, LL, and YH did the data analysis and interpretation. YH is responsible for the study conception and design. All authors contributed to the article and approved the submitted version.

## Conflict of Interest

The authors declare that the research was conducted in the absence of any commercial or financial relationships that could be construed as a potential conflict of interest.
