# Supplementary material for: Plasma β-Amyloid Levels Associated With Structural Integrity Based on Diffusion Tensor Imaging in Subjective Cognitive Decline: The SILCODE Study
Source: Front Aging Neurosci. 2021 Jan 12;12:592024. doi: 10.3389/fnagi.2020.592024 (PMC7835390; doi:10.3389/fnagi.2020.592024)
Supplement: Supplementary file 1 [file Table_1.DOCX]

Supplementary Table 1 Thresholds for memory, language and executive function tests

| Neuropsychological test | Thresholds |
| --- | --- |
| Auditory Verbal Learning Test-long delayed recall | 50-50 years old: 5  60-69 years old: 4  70-79 years old: 3 |
| Auditory Verbal Learning Test-recognition | 50-50 years old: 20  60-69 years old: 19  70-79 years old: 18 |
| Verbal Fluency Test | Junior middle school: 12  High school: 13  College: 14 |
| Boston Naming Test | Junior middle school: 19  High school: 21  College: 22 |
| Shape Trial Test A | 50-50 years old: 70s  60-69 years old: 80s  70-79 years old: 100s |
| Shape Trail Test B | 50-50 years old: 180s  60-69 years old: 200s  70-79 years old: 240s |

Thresholds for Auditory Verbal Learning Test-long delayed recall, Auditory Verbal Learning Test-recognition, Shape Trial Test A and Shape Trail Test B were stratified with age and the thresholds for Verbal Fluency Test and Boston Naming Test were stratified with education level.

Supplementary Table 2 Demographic and neuropsychological results in subjective cognitive decline (SCD) and objective cognitive impairment (OCI)

|  | SCD (n = 142) | OCI (n = 26) |
| --- | --- | --- |
| Age | 66.07±3.88 | 70.47±8.47 |
| Sex (M/F) | 45/97 | 13/13 |
| Education | 12.24±2.97 | 11.62±3.73 |
| ApoE ε4 carrier, n% | 35 (24.6%) | 12 (46.2%) |
| Hypertension, n% | 56 (39.4%) | 10 (38.5) |
| Diabetes, n% | 20 (14.1%) | 5 (19.2) |
| Hyperlipidemia, n% | 53 (37.3%) | 9 (34.6) |
| Smoking, n% | 29 (20.4%) | 7 (26.9) |
| AVLT-DR | 7.15±2.10 | 1.96±1.57 |
| AVLT-R | 22.34±1.62 | 17.72±3.08 |
| STT-A | 60.96±16.18 | 102.85±56.01 |
| STT-B | 137.04±32.87 | 235.20±111.19 |
| VFT | 18.84±4.46 | 13.92±4.77 |
| BNT | 24.96±2.86 | 21.15±5.60 |
| MMSE | 28.76±1.48 | 24.42±3.89 |
| MoCA-B | 25.67±2.33 | 19.15±4.72 |
| GDS | 2.68±2.33 | 2.76±2.60 |
| HAMA | 4.46±3.56 | 4.92±5.40 |
| HAMD | 4.17±3.91 | 5.00±4.99 |
| FAQ | 0.23±0.68 | 4.46±5.94 |

APOE, apolipoprotein E; AVLT-DR, Auditory Verbal Learning Test-delay recall; AVLT-R, Auditory Verbal Learning Test-recognition; STT-A, Shape Trail Test A; STT-B, Shape Trail Test B; VFT, Verbal Fluency Test; BNT, Boston Naming Test; MMSE, Mini-Mental State Examination; MoCA-B, Montreal Cognitive Assessment Basic Version; GDS, Geriatric Depression Scale; HAMA, Hamilton Anxiety Scale; HAMD, Hamilton Depression Scale; FAQ, Functional Activities Questionnaire.

Supplementary Table 3 Anatomical location of significant differences in white matter parameters between the high and low Aβ40 groups

|  | FA | MD |
| --- | --- | --- |
| Total number of voxels | 40037 | 58132 |
| Average values (high Aβ40 group/low Aβ40 group) | 0.608/0.625 | 0.0000756/0.0000734 |
| Corpus callosum (genu, body, splenium) | **√** | **√** |
| Corona radiata (anterior, superior, posterior) | **√** | **√** |
| Superior longitudinal fasciculus | **√** | **√** |
| Internal capsule (anterior, posterior, Retrolenticular) | **√** | **√** |
| Thalamic radiation (posterior) | **√** | **√** |
| External capsule | **√** | **√** |
| Inferior fronto-occipital fasciculus | **√** | **√** |
| Sagittal stratum | **√** | **√** |
| Cerebral peduncle | **√ ^R^** | **√** |
| Fornix (cres/stria) | **√ ^R^** | **√** |
| Tapetum | **√** | **√ ^L^** |
| Cerebellar peduncle superior |  | **√ ^L^** |
| Cerebellar peduncle middle |  | **√** |
| Cerebellar peduncle posterior |  | **√ ^L^** |
| Medial lemniscus |  | **√ ^L^** |

FA, fractional anisotropy; MD, mean diﬀusivity. Tick mark implies evidence of differences (p < 0.05, familywise error correction) between low and high Aβ40 groups. Differences were identified both left (L) and right (R) hemispheres unless otherwise stated.

Supplementary Table 4 Correlations between the average white matter parameters and neuropsychological tests

|  | FA | | MD | |
| --- | --- | --- | --- | --- |
|  | r | *p* | r | *p* |
| AVLT-DR | 0.079 | 0.354 | -0.005 | 0.951 |
| AVLT-R | 0.192 | 0.023 | -0.158 | 0.063 |
| STT-A | -0.174 | 0.041 | 0.139 | 0.102 |
| VFT | -0.049 | 0.565 | -0.081 | 0.344 |

FA, fractional anisotropy; MD, mean diﬀusivity; AVLT-DR, Auditory Verbal Learning Test-long delayed recall; AVLT-R, Auditory Verbal Learning Test-recognition; STT-A, Shape Trail Test A; VFT, Verbal Fluency Test; The analysis was adjusted for age, sex, and years of education.

Supplementary Table 5 Anatomical location of significant correlation between white matter parameters and plasma Aβ40 in SCD and OCI groups

|  | FA in SCD | MD in SCD | MD in OCI |
| --- | --- | --- | --- |
| Total number of voxels | 74177 | 98752 | 59951 |
| Average values | 0.327 | 0.000742 | 0.000758 |
| Corpus callosum (genu, body, splenium) | **√** | **√** |  |
| Forceps minor |  |  | **√** |
| Corona radiata (anterior, superior, posterior) | **√** | **√** |  |
| Superior longitudinal fasciculus | **√** | **√** | **√** |
| Inferior longitudinal fasciculus |  |  | **√** |
| Internal capsule (anterior) | **√** | **√** |  |
| Internal capsule (posterior) | **√ ^R^** | **√** |  |
| Internal capsule (retrolenticular) | **√** | **√** |  |
| Thalamic radiation (posterior) | **√** | **√** |  |
| Thalamic radiation (anterior) |  |  | **√** |
| External capsule | **√** | **√** |  |
| Inferior fronto-occipital fasciculus | **√** | **√** | **√** |
| Sagittal stratum | **√** | **√** |  |
| Cerebral peduncle | **√ ^R^** | **√** |  |
| Fornix (cres/stria) | **√ ^R^** | **√** |  |
| Tapetum | **√** | **√** |  |
| Cingulum (hippocampus) | **√** | **√** | **√ ^R^** |
| Cingulum (cingulate gyrus) |  | **√ ^R^** | **√** |
| Corticospinal tract |  | **√** | **√** |
| Cerebellar peduncle superior |  | **√** |  |
| Cerebellar peduncle middle | **√** | **√** |  |
| Cerebellar peduncle posterior |  | **√** |  |
| Medial lemniscus |  | **√** |  |

FA, fractional anisotropy; MD, mean diﬀusivity. Tick mark implies evidence of significant association between white matter parameters and plasma Aβ40 (p < 0.05, familywise error correction) in subjective cognitive decline (SCD) and patients with objective cognitive impairment (OCI) (p < 0.05, familywise error correction). Association were identified both left (L) and right (R) hemispheres unless otherwise stated.
